# Genomic Sequence of the First Porcine Rotavirus Group H Strain in the United States

**DOI:** 10.1128/genomeA.01763-15

**Published:** 2016-03-10

**Authors:** Jennifer J. Hull, Douglas Marthaler, Stephanie Rossow, Terry Fei Fan Ng, Anna M. Montmayeur, Laura Magana, Sung-Sil Moon, Baoming Jiang

**Affiliations:** a Division of Viral Diseases, National Center for Immunization and Respiratory Diseases, Centers for Disease Control and Prevention, Atlanta, Georgia, USA; b Department of Veterinary Population Medicine, University of Minnesota, St. Paul, Minnesota, USA; c SRA International, Atlanta, Georgia, USA

## Abstract

The genomic sequence of a rotavirus group H was identified in the intestine of a diarrheal pig in the United States, designated RVH/Pig-wt/USA/MN9.65/2008/GxP[x].

## GENOME ANNOUNCEMENT

Rotaviruses (RVs), which are members of the family *Reoviridae*, are a common cause of viral gastroenteritis in humans and in the young of various vertebrates (1). Rotavirus possesses an RNA genome composed of 11 double-stranded segments, encoding six structural (VPs) and five nonstructural proteins (NSPs) (1). The genome is assigned the complete descriptor of Gx-P[x]-Ix-Rx-Cx-Mx-Ax-Nx-Tx-Ex-Hx for VP7, VP4, VP6, VP1 to VP3, and NSP1-5/6, respectively, with x indicating the numbers of corresponding genotypes (2). RVs are classified into eight antigenically defined groups, rotavirus A to H (RVA to RVH) (3). RVA strains are well known for their high prevalence and pathogenesis in humans and animals, possessing either Wa-like genogroup 1 constellation (G1-P[1]-I1-R1-C1-M1-A1-N1-T1-E1-H1) or Ds-1-like genogroup 2 constellation (G2-P[2]-I2-R2-C1-M2-A2-N2-T2-E2-H2). RVB, RVC, and RVH are also known to infect humans and a limited number of animals. RVD, RVE, RVF, and RVG have been detected only in birds or pigs (2, 4). In 1997, a rotavirus strain infecting humans, adult diarrhea rotavirus (ADRV-N) (5, 6), was identified, which did not belong to previously established groups; however, it was later identified as RVH, based on VP6 sequence analysis (3). Subsequently, RVH has been detected in humans, as strain J19 in China (7), strain B219 in Bangladesh (5, 8), and in pigs from Japan (9), Brazil (10), and South Africa (11).

Here, we report the first genomic sequence for all 11 segments of a porcine RVH strain from the United States. On 16 April 2008, the University of Minnesota Veterinary Diagnostic Laboratory received three intestinal samples from 4-week-old pigs with clinical signs of diarrhea, coughing, and failure to thrive. These samples from Minnesota were homogenized together, as previously described (12), and determined to be positive for RVA, RVB, RVC, and RVH by reverse transcription-PCR (RT-PCR) (13, 14).

We used a previously described viral metagenomics approach to enrich and sequence viral nucleic acids, followed by MiSeq and *de novo* assembly of the resulting reads (15). Briefly, viral RNA was reversely transcribed and amplified using a random primer. A next-generation sequencing (NGS) library was prepared, using the Nextera XT kit; sequencing followed using Illumina’s MiSeq platform. A total of 766,560 reads were generated, *de novo* assembled, and analyzed in the bioinformatics pipeline, as described previously (16).

We obtained the following near-complete sequences for RVH strain MN9.65: for VP1 (segment 1, 3,369 bp); VP2 (segment 2, 2,977 bp); VP3 (segment 3, 1,167 bp); VP4 (segment 4, 1,275 bp); NSP1 (segment 5, 1,248 bp), the only segment containing the entire coding region; NSP3 (segment 7, 408 bp); NSP2 (segment 8, 913 bp); VP7 (segment 9, 456 bp); NSP4 (segment 10, 687 bp); and NSP5/6 (segment 11, 493 bp). The VP6 gene was deposited previously (14). This RVH strain MN9.65 shares 88% (VP1), 90% (VP2), 90% (VP3), 88% (VP4), 82% (VP7), 88% (NSP1), 90% (NSP2), 94% (NSP3), and 85% (NSP4) nucleotide identity with porcine RVH strain SKA-1 from Japan. Obtaining all 11 segments of RVH will facilitate further rotavirus H surveillance in pigs globally and allow for the development of improved screening methods for RVH strains.

### Nucleotide sequence accession numbers.

The sequences for RHV/MN9.65/2008 have been deposited in GenBank under accession numbers KU254582 to KU254592.
